# Stromal Liver Kinase B1 [STK11] Signaling Loss Induces Oviductal Adenomas and Endometrial Cancer by Activating Mammalian Target of Rapamycin Complex 1

**DOI:** 10.1371/journal.pgen.1002906

**Published:** 2012-08-16

**Authors:** Pradeep S. Tanwar, Tomoko Kaneko-Tarui, LiHua Zhang, Yoshihiro Tanaka, Christopher P. Crum, Jose M. Teixeira

**Affiliations:** 1 Vincent Center for Reproductive Biology, Department of Obstetrics, Gynecology, and Reproductive Biology, Massachusetts General Hospital, Boston, Massachusetts, United States of America; 2 School of Biomedical Sciences and Pharmacy, University of Newcastle, Callaghan, New South Wales, Australia; 3 Division of Women's and Perinatal Pathology, Department of Pathology, Brigham and Women's Hospital, Harvard Medical School, Boston, Massachusetts, United States of America; SA Pathology, Australia

## Abstract

Germline mutations of the Liver Kinase b1 (*LKB1/STK11*) tumor suppressor gene have been linked to Peutz-Jeghers Syndrome (PJS), an autosomal-dominant, cancer-prone disorder in which patients develop neoplasms in several organs, including the oviduct, ovary, and cervix. We have conditionally deleted *Lkb1* in Müllerian duct mesenchyme-derived cells of the female reproductive tract and observed expansion of the stromal compartment and hyperplasia and/or neoplasia of adjacent epithelial cells throughout the reproductive tract with paratubal cysts and adenomyomas in oviducts and, eventually, endometrial cancer. Examination of the proliferation marker phospho-histone H3 and mammalian Target Of Rapamycin Complex 1 (mTORC1) pathway members revealed increased proliferation and mTORC1 activation in stromal cells of both the oviduct and uterus. Treatment with rapamycin, an inhibitor of mTORC1 activity, decreased tumor burden in adult *Lkb1* mutant mice. Deletion of the genes for Tuberous Sclerosis 1 (*Tsc1*) or *Tsc2*, regulators of mTORC1 that are downstream of LKB1 signaling, in the oviductal and uterine stroma phenocopies some of the defects observed in *Lkb1* mutant mice, confirming that dysregulated mTORC1 activation in the *Lkb1*-deleted stroma contributes to the phenotype. Loss of PTEN, an upstream regulator of mTORC1 signaling, along with *Lkb1* deletion significantly increased tumor burden in uteri and induced tumorigenesis in the cervix and vagina. These studies show that LKB1/TSC1/TSC2/mTORC1 signaling in mesenchymal cells is important for the maintenance of epithelial integrity and suppression of carcinogenesis in adjacent epithelial cells. Because similar changes in the stromal population are also observed in human oviductal/ovarian adenoma and endometrial adenocarcinoma patients, we predict that dysregulated mTORC1 activity by upstream mechanisms similar to those described in these model systems contributes to the pathogenesis of these human diseases.

## Author Summary

Peutz-Jeghers Syndrome patients have autosomal dominant mutations in the *LKB1/STK11* gene and are prone to developing cancer, predominantly in the intestinal tract but also in other tissues, including the reproductive tracts and gonads. To elucidate the mechanisms disrupted by the loss of LKB1 in the reproductive tract, we have developed a mouse model with deletion of *Lkb1* specifically in stromal cells of gynecologic tissues. These mice show stromal cell expansion and develop oviductal adenomas and endometrial cancer. Deletion of either *Tsc1* or *Tsc2* genes, which are mutated in patients with Tuberous Sclerosis Complex and whose protein products are indirect downstream targets of LKB1 signaling, resulted in some of the same defects observed in *Lkb1* mutant mice. Activation of mammalian Target Of Rapamycin Complex 1 (mTORC1), a common effector of disrupted LKB1, TSC1, and TSC2 signaling, was observed in all mutant tissues examined, suggesting that uninhibited mTORC1 activity is necessary for the phenotypes. Suppression of mTORC1 signaling by rapamycin reduced tumor burden in *Lkb1* mutant mice, confirming the link between dysregulation of mTORC1 to development of the *Lkb1* mutant phenotype and suggesting that therapeutic targeting of LKB1/TSC1/TSC2/mTORC1 signaling would benefit human Peutz-Jeghers Syndrome and Tuberous Sclerosis patients with reproductive tract disease.

## Introduction

The embryonic Müllerian ducts, which are composed of a simple columnar epithelium surrounded by mesenchymal cells, differentiate into the oviducts, uterus, cervix, and anterior portion of the vagina [1]. During differentiation, epithelial-mesenchymal communication plays an important role in specification of the Müllerian duct epithelium into ciliated and secretory (oviduct), columnar (uterus), and squamous (cervix) epithelium [2]. Confirmation of the control exerted by the stroma on differentiation, shown using tissue recombination studies mixing uterine or vaginal stroma and epithelia, have revealed that the fate of epithelial cells depends on stromal/mesenchymal signaling [2], [3]. In the uterus, epithelial-mesenchymal crosstalk also plays an important role in development of epithelial cancer. For example, in our recent study, we showed that conditional deletion of Adenomatous Polyposis Coli (APC) in endometrial stromal cells results in their conversion to a myofibroblast phenotype, which was sufficient to initiate endometrial hyperplasia that could lead to endometrial cancer in mice [4]. The physiological relevance of the endometrial stroma cell conversion was confirmed when a myofibroblast stromal phenotype was also observed in human endometrial epithelial cancer patient tissue samples [4].

Peutz-Jeghers syndrome (PJS) is a hereditary cancer-prone disorder linked to mutation of *LKB1* (also known as Serine/Threonine Protein Kinase 11; *STK11*) [5]. Patients with PJS are at high risk of developing cancerous lesions in various organs including testis, ovary, endocervix, breast, and colon [6]. *LKB1* encodes an evolutionarily conserved serine/threonine kinase that phosphorylates and activates a family of related AMP kinases (AMPK) in response to a decline in the cellular ATP:AMP ratio, acting as a metabolic rheostat to maintain energy homeostasis [7]. One of the best studied targets of AMPK is mammalian target of rapamycin complex 1 (mTORC1), a master regulator of proliferation, which is inhibited indirectly by maintaining the TSC1/TSC2 tumor suppressor complex and directly by phosphorylation of regulator-associated protein of mTOR (Raptor), a substrate binding component of the rapamycin-sensitive mTORC1 [5]. LKB1-AMPK tumor suppressor activity has also been associated with another of its major functions, controlling cell polarity, which appears to be the central mechanism suppressing tumorigenesis in a pancreatic cancer model with loss of LKB1 [8].

Adenoviral-delivered Cre-mediated deletion of *Lkb1* in murine endometrial epithelium can drive development of highly invasive endometrial adenocarcinoma [9] showing that LKB1 is an important tumor suppressor in endometrial carcinogenesis. Mutations in *LKB1* are also detected in human cervical cancer patients, and PJS patients also develop endocervical cancer known as adenoma malignum/minimum deviation of adenocarcinoma [10]. Although most reports of tumorigenesis are from mutated epithelial cells, loss of LKB1 in mesenchymal cells using Smooth Muscle 22-cre has been shown to lead to the development of polyps with features similar to those found in PJS patients [11]. Of note, gastrointestinal polyps from PJS patients contain more myofibroblastic stromal cells, which is similar to the phenotype mice develop after deletion of endometrial stromal APC [4]. LKB1 is highly expressed in the mesenchymal cells of human gonads and patients with PJS develop ovarian and testicular stromal tumors [12]–[14], suggesting that LKB1 might be an important tumor suppressor in the stromal cells of reproductive organs. This hypothesis is supported by the recent findings that, whereas, heterozygous deletion of LKB1 in both epithelium and stroma results in endometrial cancer [9], heterozygosity of LKB1 in the epithelium alone does not [15].

In order to investigate whether deletion of LKB1 from reproductive tract and gonadal stromal cells leads to the gynecological abnormalities observed in PJS patients, we generated mice with conditional deletion of LKB1 in the stromal cells of the female reproductive tract using Müllerian inhibiting substance receptor 2-driven Cre (Misr2-Cre) [16]. A significant proportion of PJS patients harbor mutations that encompass whole or partial *LKB1* gene deletions, make this mouse model appropriate for studying this disease. We observed expansion of the myofibroblast population accompanied by hyperplasia and/or neoplasia of the epithelia of both oviduct and uterus. Loss of TSC1 or TSC2 using the same Cre is able to phenocopy some of the reproductive pathologies of *Lkb1*-deleted mice, suggesting that their common downstream target, mTORC1, plays a role in their pathogenesis when dysregulated. These results show the importance of mesenchymal LKB1/TSC1/TSC2/mTORC1 signaling in the female reproductive tract and provide a compelling rationale for considering the therapeutic option of mTORC1 inhibitors for these patients.

## Results

Women with PJS are prone to developing multiple gynecological pathologies including oviductal and uterine cancers [5], [6], [17] and minimal deviation adenocarcinoma/adenoma malignum of uterine cervix [18]. While these latter tumors are malignant in nature, histologically they appear as benign tumors because of the significant amount of stroma and sparsely placed, normal looking epithelial glands [18]. However, the pathogenesis of female reproductive tract abnormities and the mechanisms disrupted by mutant *LKB1* are still unclear. In this study, we have developed conditional deletion of exon 3–6 of the *Lkb1* gene in the Müllerian duct mesenchyme-derived stromal cells of the murine female reproductive tract by crossing Müllerian inhibiting substance type 2 receptor-drive Cre (Misr2-Cre, also known as antiMüllerian hormone type 2 receptor Amhr2-Cre) [16] mice with *Lkb1^fl/fl^* [19] mice to develop *Lkb1^cko^* mice to determine whether the loss of LKB1 affects either female reproductive development or results in PJS-associated pathologies. Previous studies have successfully used Misr2-Cre to induce recombination in mesenchymal cells of the female reproductive tract [4], [20]–[23]. To confirm that Misr2-Cre expression is limited to the mesenchymal cells of the female reproductive tract, we used the *Rosa26-Lacz* Cre reporter strain in which β-galactosidase is expressed in cells also expressing Misr2-Cre and showed that, in the oviducts, cervix and vagina, β-galactosidase activity is limited to the stromal cells (Figure S1A). In the uterus, β-galactosidase activity is confined to the mesenchymal cells as previously described [4]. Genomic PCR confirms that recombination of the *Lkb1* flox allele occurs in the reproductive tract tissues of mutant mice (Figure S1D).

### Abnormal expansion of the stromal compartment in Lkb1^cko^ oviducts

**Figure 1:**
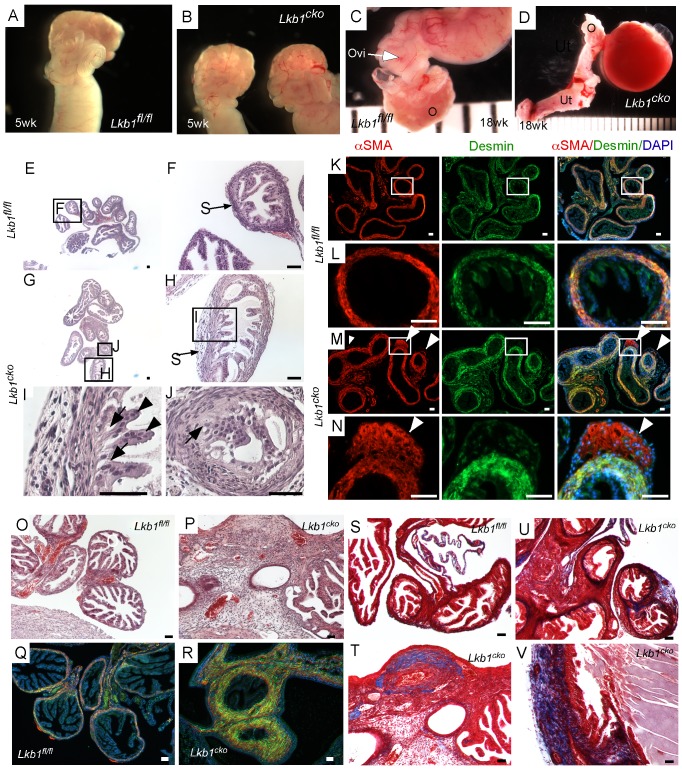
Oviductal abnormalities in *Lkb1^cko^* mice. Gross examination of 5 week old control (A) and *Lkb1^cko^* mutant (B) mice revealed normal looking coiled oviducts. Oviducts of 18 week old control (C) and mutant (D) mice show the presence of cysts, which were either large as shown in Panel D or microscopic (not shown). Histology of 5 week old control (E, and higher magnification of boxed area F) and mutant (G–J) oviducts with abnormal epithelial and stromal cells. Higher magnification images of oviducts showing abnormal epithelium next to mutant stromal cells (H and J; arrow); normal looking epithelial cells (I; arrowhead) were present away from the stromal cells. Colocalization of desmin and αSMA in 5 week old (n = 3) control (K–L) and mutant (M–N) oviducts. White arrowheads mark abnormal cell growth in mutant oviducts. Histology of 18 week old control (O) and mutant (P) oviducts showed greater stromal expansion in the mutants, which was confirmed by co-immunostaining with desmin and αSMA of adult control (Q) and mutant (R) oviducts. Masson's Trichrome staining indicates significantly greater collagen deposition (blue) in 18 week old mutant oviducts (T–V) compared to controls (S). Ovi; oviducts; O; ovary, Ut; uterus. Bars: 50 um.

No gross defects were observed in 5 week old control and *Lkb1^cko^* mutant (N = 3/each) oviducts (Figure 1A and 1B). After 18 weeks, grossly visible cystic growths of various sizes were observed in the oviducts of mutant females (N = 5/5; Figure 1D). Normal oviduct development was observed in age-matched control littermates (N = 5/5; Figure 1C). Initial examination of ovaries from both groups of mice revealed the presence of corpora lutea, suggesting normal ovulation and ovarian functions (data not shown).

Histological examination of the control and mutant oviducts was performed to evaluate cellular changes after loss of LKB1. At 5 weeks of age, H&E-stained sections of mutant oviducts showed increased thickness of the LKB1-deleted stromal compartment compared with controls (N = 3; Figure 1E–1J). Stromal cells of the mutant oviducts were also more disorganized compared with age-matched controls and oviductal epithelial cells of mutant mice had a more irregular and vacuolated arrangement (Figure 1G–1J) and were often found occluding the lumen (Figure 1J). Examination of the mesenchymal/smooth muscle cell markers, desmin and αSmooth Muscle Actin (αSMA) in the 5 week old oviducts by immunofluorescence confirmed expansion of αSMA+/desmin- or weakly desmin+ stromal cell population, indicating dysregulated proliferation of *Lkb1*-deleted stromal cells (Figure 1M and 1N) compared with controls (Figure 1K and 1L). A similar expansion of αSMA+/desmin- myofibroblast cells was also observed in polyps from human PJS patients and other mouse models with loss of LKB1 [11]. By 18 weeks, histological examination of mutant oviducts showed massive expansion of the stromal compartment compared with controls (Figure 1O and 1P). Co-staining with αSMA and desmin confirmed the increase in smooth muscle/myofibroblast cells in the stroma of *Lkb1^cko^* oviducts (Figure 1Q and 1R).

The extracellular matrix (ECM) plays an important role in determining epithelial cell integrity and polarity [24]. In the uterus, stromal cells secrete and deposit various components of ECM [25], including collagen and laminin, which play an important role in endometrial remodeling [26]. Additionally, altered production of ECM components is observed in various endometrial pathologies [27], [28]. For this study, we hypothesized that expansion of the stromal population in mutant oviducts affects the production and deposition of ECM components. To test this we performed Masson's Trichrome staining of control and mutant oviducts (N = 3/each) and observed a significant increase in blue staining, indicative of collagen in the stromal compartment of mutant oviducts (Figure 1U–1V). By comparison, collagen-specific staining is limited to the basement membrane of the control oviducts (Figure 1S). Next we examined expression of various cytoskeleton proteins (cytokeratin, β-catenin, E-cadherin and Tight Junction Protein-1/Zona Occuldens1 (TJP1/ZO1) to determine if there were any changes in oviductal epithelial cells due to the stromal expansion and alterations in ECM (Figure S2). No differences in the expression of cytokeratin, β-catenin, E-cadherin, and TJP-1 were observed between attached mutant and control oviductal epithelial cells. However, their expression did appear abnormal in detached epithelial cells present in the lumen of the mutant oviducts.

To confirm that the normal differentiation of the oviductal epithelial cells was not affected by the changes in the mesenchymal cells of the mutant oviducts, we perform colocalization of paired box gene 2 (PAX2) and αSMA in oviducts of both control and mutant mice (Figure S2). PAX2 is a marker of the murine reproductive tract epithelial cells and loss of the *Pax2* gene is associated with dysgenesis of reproductive tract [29]. Normally PAX2 is not expressed in the epithelium of the distal segment of the mouse oviduct and no changes in the expression pattern of PAX2 were observed between control and mutant oviducts (Figure S2).

### Abnormal cystic growth and hyperplasia of Lkb1^cko^ oviductal epithelium

**Figure 2:**
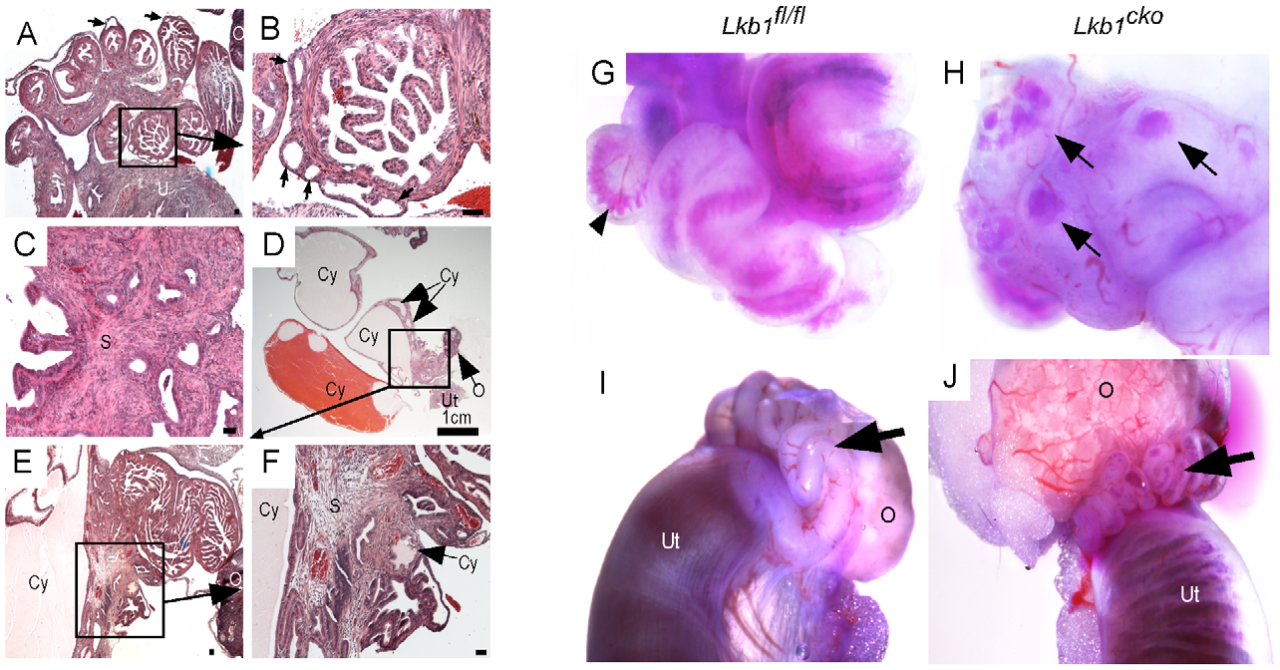
Hyperplastic and/or cystic growth in oviducts of *Lkb1^cko^* mice. (A and B) Abnormal epithelial growth (arrow) in adult mutant oviducts. Panel B is a magnified image of the area outlined by a solid rectangle in panel A. Glandular epithelium is present in some parts of the mutant oviduct (C). Cysts of variable size were observed in adult mutant mice (D–F). Blue dye injections into the oviductal lumen (G and H) and from the lumen of the uterus (I and J) in control and mutant mice. Arrowhead in panel G marks blue dye in the oviductal lumen. Arrows in Panel H point to accumulation of dye in oviductal cysts. Arrows in panels I and J are directed to the oviducts. O: ovary, S: stroma, Cy: cyst, Ut: uterus. Bars: 50 um unless otherwise indicated.

PJS patients have been diagnosed with cystic growths in their Fallopian tubes that are either filled with translucent white fluid or pus (pyosalpinx) [17]. Figure 1D shows a typical large cyst in the *Lkb1^cko^* oviduct, and histological examination of *Lkb1^cko^* mutant oviducts revealed numerous smaller cystic growths as well (N = 5/5) (Figure 2). The distended blind cysts in mutant mice were highly variable in size, often accompanied by stromal expansion (Figure 2C), and often filled with either bloody or pale fluid similar to hematosalpinx, and pyosalpinx, respectively (Figure 2D). We injected bromophenol blue dye into the lumen of mutant and control oviducts to determine whether the fluid accumulating in the cysts was derived from the lumen, which in normal oviducts should pass straight through [20]. In mutant oviducts, abnormal accumulation of dye in small cystic growths was observed, suggesting that the cysts were directly connected to the lumen or that the oviductal epithelial basement membrane had become permeable (Figure 2G and 2H). Additionally, when dye was injected from the uterine side, no dye was observed in the oviductal lumen of control mice but was observed in the mutant oviducts (Figure 2I and 2J), suggesting a defective uterotubular junction in mutants and consistent with loss of control of retrograde flow [20].

**Figure 3:**
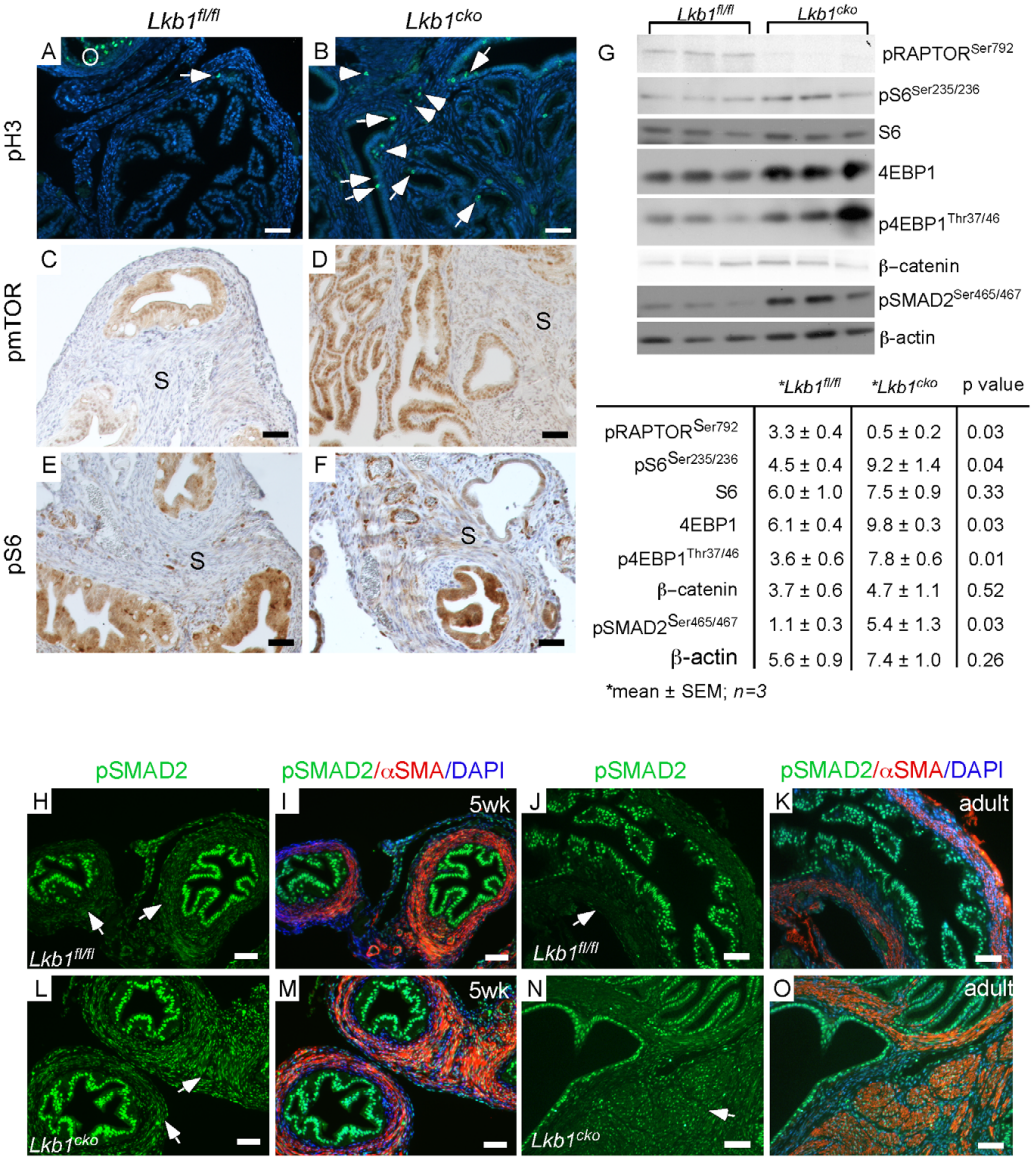
Activation of mTORC1 and TGFβ signaling in the stromal compartment of mutant oviducts. Expression analysis of pH 3, a marker for proliferation, revealed increased numbers of dividing cells in the stromal (arrowhead) and epithelial (arrow) compartments of mutant oviducts (B) compared with controls (A). pH 3 staining in antral follicles of the ovary (O) was used as a positive control (A). Phosphorylation of mTOR (C and D) and S6 (E and F) is increased in the stromal cells of the mutants (D and F). (G) Western blot analyses of pRAPTOR (Ser792), pS6 (Ser235/236), S6, 4EBP1, p4EBP1 (Thr37/46), β-catenin, pSMAD2 (Ser465/467) oviduct protein extract in mutants and controls. β-actin was used as loading control. (H–O) pSMAD2 protein expression was also increased in mutant (L, M, N and O) compared to control oviducts (H, I, J and K). Bars: 50 um.

LKB1 plays an important role in various biological processes, including cell proliferation, by interacting with AMP kinases (AMPKs) and regulating mTORC1 activation [5]. We therefore examined expression of phospho-histone H3 (pH 3), a marker for mitotic cells, to determine the rate of cellular proliferation in control and mutant oviducts. Increased pH 3-positive cells in both epithelial (arrow) and stromal (arrowheads) compartments was observed in mutant oviducts compared to controls (Figure 3A and 3B). Analysis of the expression of phosphorylated forms of mTOR and riboprotein S6, a target of S6 Kinase that is downstream of activated mTORC1, showed increased phosphorylation of both mTOR (Ser2448) and S6 (Ser235/236) in the mesenchymal cells of the mutant oviducts (Figure 3C–3F). Since phosphorylation of S6 at Ser235/236 sites is regulated by both mTORC1 and mitogen-activated protein kinases [30] we confirmed mTORC1 involvement by western blot analyses and showed decreased expression of pRAPTOR (Ser792) and increased phosphorylation of S6 (Ser235/236; pS6/S6 ratio: 12.3±1.9 mutant/8.2±1.9 control) and eukaryotic translation initiation factor 4E-Binding Protein 1 (4EBP1, Thr37/46; p4EBP1/4EBP1 ratio: 0.8±0.0 mutant/0.5±0.1 control) in the mutant oviducts (n = 3) (Figure 3G). LKB1 has also been shown to affect Wnt signaling, dysregulation of which causes defects in oviduct development and differentiation [5], [22], [31]. We measured β-catenin protein levels in oviducts by western blot and observed no change in expression of β-catenin between control and mutant oviducts (Figure 3G), suggesting that defects observed in *Lkb1* mutant oviducts are independent of canonical Wnt signaling.

LKB1 is also an inhibitor of transforming growth factor β (TGFβ) signaling [32], which can play an important role in fibroblast differentiation and fibrosis [33] and has been implicated in carcinogenesis of various organs [4], [34], [35]. In this study, we evaluated TGFβ signaling and observed an increase in expression and nuclear translocation of the phosphorylated and active form (Ser465/467) of its downstream target, SMA and Mothers Against Decapentaplegic homolog 2 (SMAD2), in mesenchymal cells of both 5 week-old and adult mutant oviducts compared to controls (Figure 3H–3O), which was confirmed by western blot analysis (Figure 3G). Since stromal bone morphogenetic protein (BMP) signaling also plays a role in regulating the growth of intestinal epithelium and its inhibition leads to polyposis in mice [36], we examined and found comparable pSMAD1/5/8 in *Lkb1* mutant and control mice (data not shown), suggesting BMP signaling is not affected in our model system. These results suggest that stromal expansion and increased ECM deposition in the mutant oviducts could both be the result of dysregulated TGFβ signaling.

### Loss of TSC1 or TSC2 in oviductal stromal cells causes development of cystic and abnormal growth in mutant oviducts

**Figure 4:**
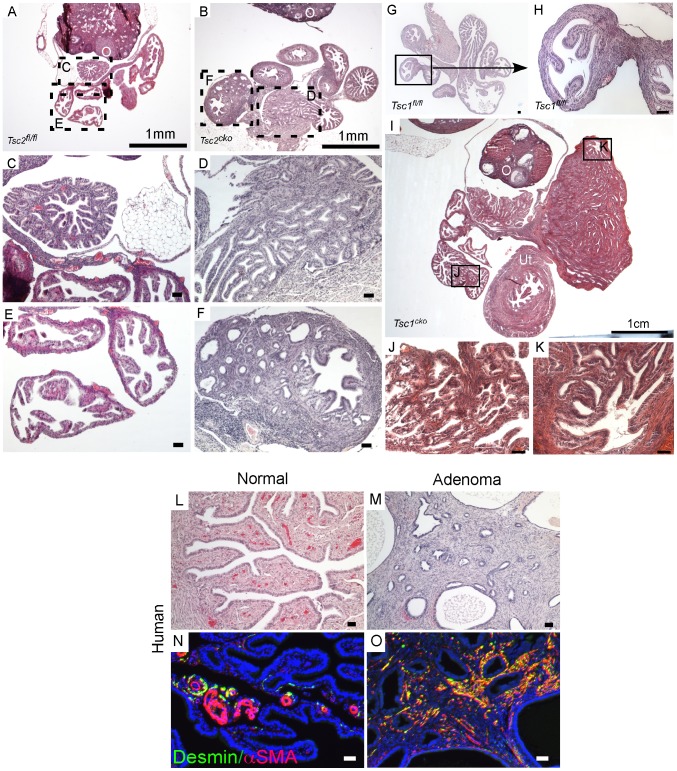
Conditional deletion of *Tsc1* or *Tsc2* phenocopies some of the changes observed in *LKB1^cko^* oviducts. H&E stained adult control (A, C and E) and *Tsc2^cko^* mutant (B, D and F) oviducts. Panel C to F are high magnification images of rectangular areas mark with dotted line in Panel A and B. (G and H) Histology of control oviducts. (I–K) Abnormal growth containing epithelial glands and stroma in oviducts of older *Tsc1^cko^* mice. Panels J and K are higher magnification images of areas outlined by solid rectangles in Panel I. Oviducts from normal human (L) and patients with adenomas (M). Increased numbers of desmin- and αSMA-positive cells in the stromal compartment in oviductal adenoma patients (O) compared to normal oviducts (N). O: ovary; Ut: uterus. Bars: 50 um unless otherwise indicated.

Because we observed increased mTORC1 activity in the stromal cells of *Lkb1^cko^* oviducts (Figure 3), we reasoned that deletion of other genes involved in the regulation of the mTORC1 pathway would phenocopy the defects observed in *Lkb1^cko^* oviducts. For example, TSC1 and TSC2 act together as a complex to modulate the activity of the mTORC1 signaling pathway and loss of TSC1 or TSC2 leads to hyperactivation of mTORC1 signaling [37]. To study the effects of TSC1 and TSC2 loss in mesenchymal cells of the oviduct, we developed mice with conditional deletion of *Tsc1* (*Tsc1^cko^*) and *Tsc2* (*Tsc2^cko^*) by using the same Misr2-Cre we used to generate the *Lkb1^cko^* mice. Deletion of *Tsc2* in the female reproductive tract (oviduct, ovary, and uterus) was confirmed using genomic PCR (Figure S1E). Gross examination of female reproductive tract revealed no obvious differences between control and *Tsc2^cko^* mutant mice (data not shown). However, histological examination of 6 week old oviducts revealed moderate hyperplasia of epithelial cells in mutants compared with controls (Figure S3). By 18 weeks, the oviducts of *Tsc2^cko^* mice showed expansion of both stromal and epithelial compartments accompanied by abnormal cystic growth (N = 5/5) (Figure 4B, 4D, 4F) compared to controls (N = 4/4) (Figure 4A, 4C, 4E). Similar to *Lkb1^cko^* and *Tsc2^cko^* mutants, abnormal cystic lesions were also observed in the oviducts of the older *Tsc1^cko^* mutant females (N = 3/each) (Figure 4I–4K).

Histological examination of the oviducts from *Lkb1^cko^, Tsc1^cko^ and Tsc2^cko^* mice also revealed the presence of adenomyomas (Figure 2 and Figure 4), nodular lesions consisting of simple glandular, often hyperplastic epithelium with abnormal stromal cell expansion as a prominent feature (Figure 1 and Figure 4). In humans, adenomyomas are observed in various organs including the oviduct, ovary, uterus, and intestine [38]–[41]. Because we observed development of adenomyomas in the mutant animals with a significant myofibroblastic stromal cell population, we hypothesized that human oviductal/ovarian adenomyomas might also be the result aberrant stromal cell expansion and conversion to myofibroblasts. We analyzed expression of αSMA and desmin in normal human oviducts and oviductal adenomas to study changes in the stromal compartment (Figure 4L–4O) and found that expression of these markers was limited to the blood vessels and very little staining was observed in the stromal cells (Figure 4N). In contrast, αSMA and desmin staining is increased in the stroma of the oviductal adenomyomas (N = 8/10) (Figure 4O), suggesting of expansion of myofibroblast/smooth muscle cell population.

### Deletion of Lkb1/Tsc2/Tsc1 in the endometrial stromal compartment is sufficient to initiate uterine epithelial hyperplasia and neoplasia

**Figure 5:**
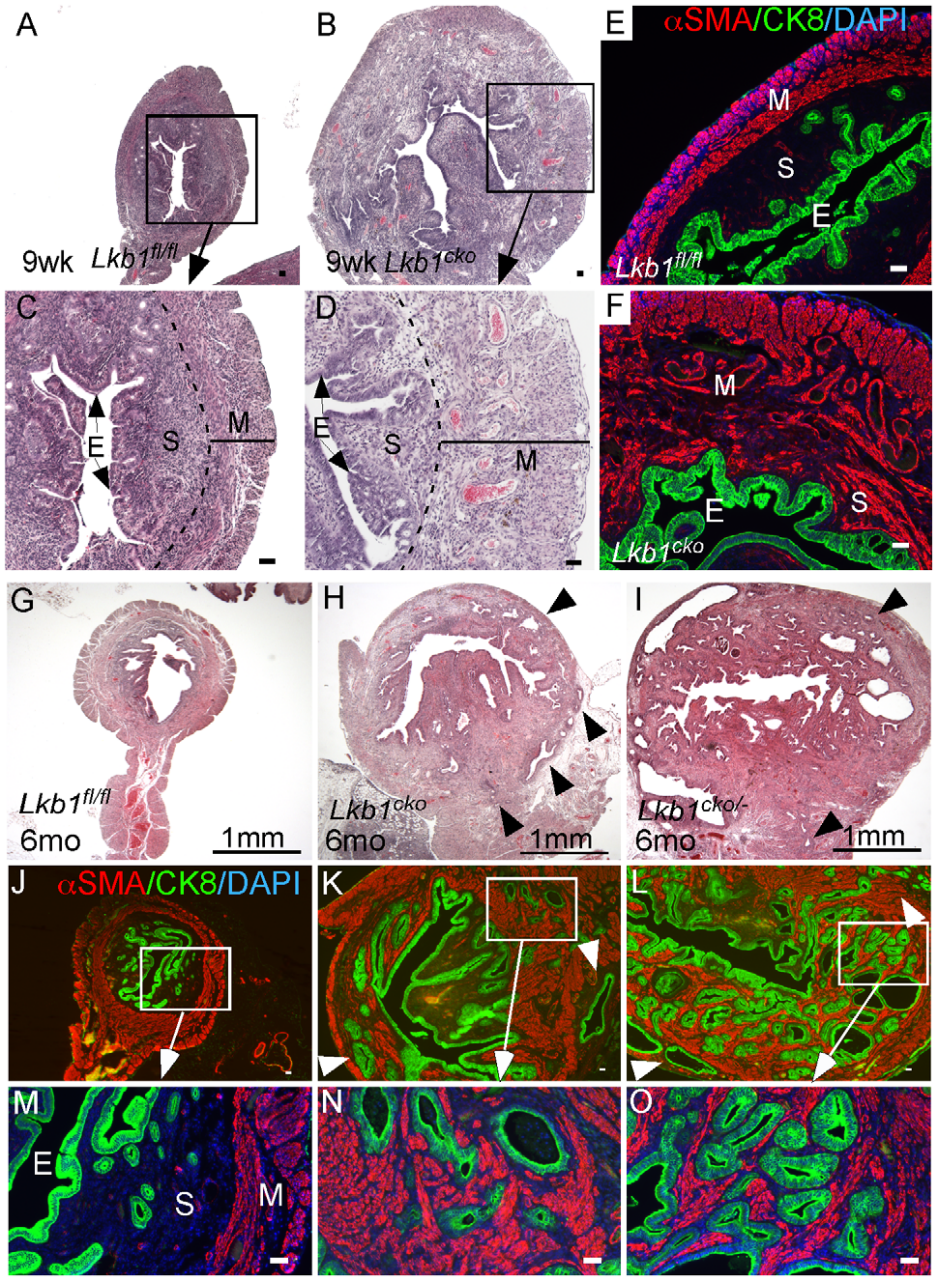
Loss of LKB1 in mesenchymal cells induces endometrial cancer. H&E-stained sections of 9 week old control (A and C) and *Lkb1^cko^* mutant (B and D) uteri. Immunostaining for CK8 (green) and αSMA (red) of 9 week old control (E) and *Lkb1^cko^* (F) uteri. Histology and CK8/αSMA immunostaining of 6 month old *Lkb1^fl/fl^* (G, J and M), *Lkb1^cko^* (H, K and N), *Lkb1^cko/-^* (I, L and O). Arrowheads in panels H, I, K, L point to invasive glandular epithelium present in the myometrial compartment, a hallmark of endometrial adenocarcinoma. E: epithelium, M: myometrium, S: stroma. Bars: 50 um.

The mouse uterus is comprised of three different compartments: endometrial (luminal and glandular) epithelium, endometrial stroma, and myometrium (smooth muscle cells) [42]. In our previous study, we showed that deletion of APC in the stromal compartment is sufficient to induce endometrial cancer in mice and also observed comparable changes in human endometrial cancer patients suggesting that mesenchymal cells play an important role in the etiology of endometrial cancer [4]. The LKB1/mTORC1 signaling pathways have also been implicated in endometrial carcinogenesis [15], [43]. We examined uteri from 9 week old control and *Lkb1^cko^* mutant mice and found evidence of endometrial epithelial hyperplasia and endometrial cancer with expansion of the myofibroblastic population in the *Lkb1^cko^* mice but not in the controls (Figure 5A–5D). Colocalization of αSMA and cytokeratin 8 (CK8; epithelial marker) confirmed expansion of myofibroblast cell population in the stromal compartment adjacent to the CK8+ epithelial lining in mutant uteri (Figure 5F and Figure S4). In contrast, only αSMA-negative stromal cells are present close to CK8+ epithelial cells in control uteri (Figure 5E).

Examination of the reproductive tracts from older mutant animals (N = 5/5) showed that their uteri were greatly enlarged (Figure S4B) and that endometrial epithelial glands had invaded the myometrial compartment, which is a hallmark of endometrial adenocarcinoma [44] (Figure 5H, 5K, 5N). In contrast, control uteri (N = 5/5) were much smaller and epithelial cells were limited to the endometrial/stromal compartment (Figure 5G, 5J, 5M; Figure S4A). A previous study showed that approximately 30% of *Lkb1+/−* mice developed endometrial cancer and that biallelic uterine epithelial specific loss of *Lkb1* using adenoviral Cre was sufficient to induce highly invasive endometrial adenocarcinoma [9]. To compare tumorigenesis in mice with stromal *Lkb1^cko^* alone with mice with epithelial loss of *Lkb1*, we generated another mouse (*Lkb1^cko/-^*) in which one allele of *Lkb1* is deleted in all cells, including the epithelial cells of the uterus, and the other allele is floxed only in the Müllerian duct mesenchyme-derived stromal cells (Figure 5I, 5L, 5O; Figure S4C). Comparison of endometrial cancer formed in *Lkb1^cko^* and *Lkb1^cko/-^* using αSMA and CK8 immunostaining revealed that tumors in both model systems were very similar (Figure 5N and 5O). Particularly noteworthy was the increased αSMA+ cell population adjacent to CK8+ cells observed in both models (Figure 5N and 5O) compared with controls. However, tumors formed in *Lkb1^cko/-^* were bigger, more aggressive and invasive compared to *Lkb1^cko^* mice, supporting the findings of the previous report [9] and showing the importance of epithelial LKB1 loss to endometrial carcinogenesis. Similar to *Lkb1^cko^* oviducts (Figure 3), we observed increased proliferation of uterine mesenchymal cells as confirmed by pH 3 staining in *Lkb1^cko^* mutants compared with controls (Figure S4F and S4G).

**Figure 6:**
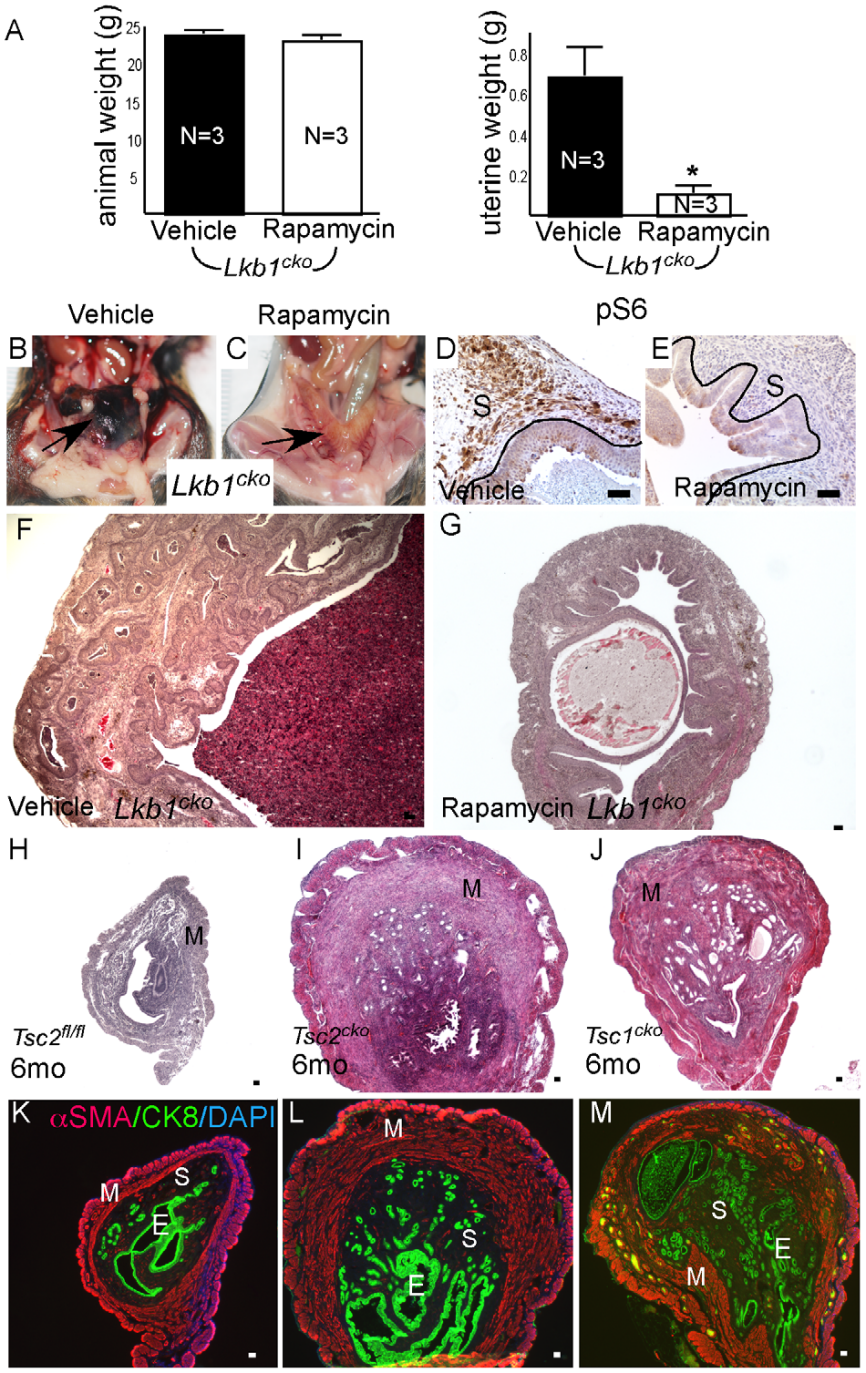
Dysregulated mTORC1 activity in *Lkb1* mutant mice. (A) Animal and uterine weight of *Lkb1^cko^* mice (>7 month old mice) treated with rapamycin or vehicle control. (B and C) Gross female reproductive tracts (arrow) of vehicle- and rapamycin-treated *Lkb1^cko^* mice. pS6 IHC (D and E) and histology (F and G) of *Lkb1* mutant mice treated with vehicle or rapamycin. The black lines in panels D and E demarcate stromal (S) from epithelial cells. Histology and CK8/αSMA staining of *Tsc2^fl/fl^* (H and K), *Tsc2^cko^* (I and L), and *Tsc1^cko^* (J and M) uteri. Bars: 50 um.

Examination of pmTOR, pS6, and pSMAD2 expression revealed increased mTORC1 and TGFβ signaling activity in *Lkb1^cko^* uteri compared to controls (Figure S4H–S4M). Because we observed increased mTORC1 activity in *Lkb1^cko^* uteri, we treated aged *Lkb1^cko^* mice (>7 month old) with rapamycin or vehicle (N = 3/group) as previously described [45]. After 3 weeks of treatment, we observed decreased uterine weight accompanied by reduced expression of pS6 in rapamycin-treated mice compared with controls (Figure 6A–6E). Histological examination of rapamycin- and vehicle-treated uteri and oviducts showed suppression of endometrial carcinogenesis (Figure 6F and 6G) and inhibition of cyst formation (Figure S5A), respectively, with rapamycin treatment confirming the involvement of mTORC1 in the pathogenesis of the *Lkb1* mutant phenotype.

We also analyzed uteri from *Tsc1^cko^* and *Tsc2^cko^* mice to determine whether deletion of these upstream regulators of mTORC1 activity resulted in phenotypes similar to those observed in the *Lkb1^cko^* uteri. Compared to controls, *Tsc2^cko^* and *Tsc1^cko^* uteri were enlarged and showed hyperplasia or neoplasia of the endometrial epithelium (Figure 6H–6J). Immunolocalization of αSMA and CK8 revealed that, similar to *Lkb1* mutant uteri, *Tsc2^cko^* and *Tsc1^cko^* uteri showed expansion of the αSMA+ and CK8+ cell populations (Figure 6K–6M). In contrast to *Lkb1^cko^* or *Lkb1^cko/-^* endometrial cancers, myometrial invasion of epithelial cells (Figure 6L and 6M) and squamous metaplasia of endometrial epithelium (Figure S5B) was not observed in either *Tsc2^cko^* or *Tsc1^cko^* uteri.

### Synergistic effect of Pten deletion on Lkb1^cko^ phenotype

**Figure 7:**
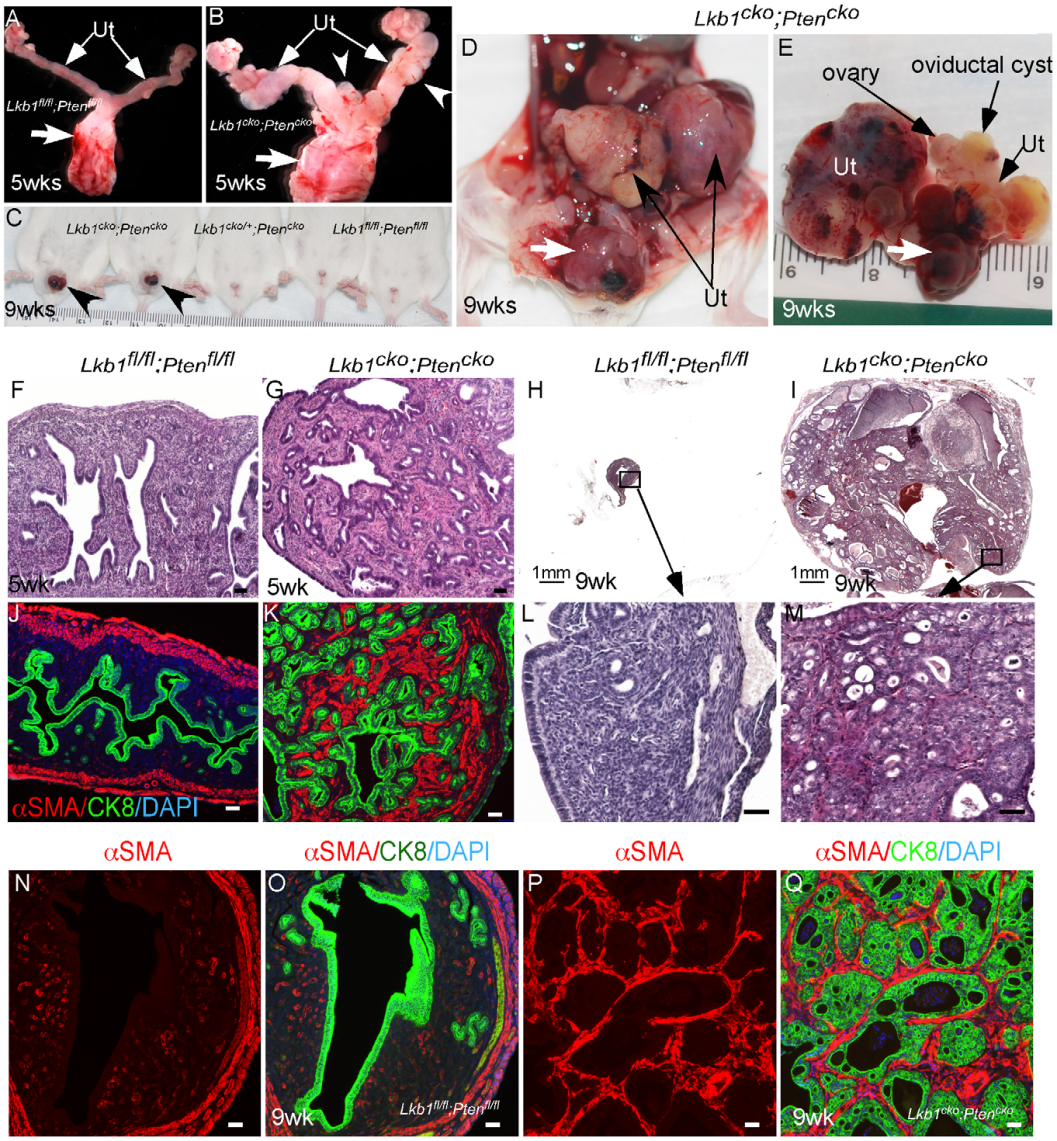
Formation of highly aggressive endometrial adenocarcinoma in *Lkb1^cko^;Pten^cko^* mice. (A and B) Female reproductive tracts from *Lkb1^fl/fl^;Pten^fl/fl^* and *Lkb1^cko^;Pten^cko^* mice. Arrowheads point to overgrowths. Arrows: Cervix and vagina. (C) *Lkb1^cko^;Pten^cko^* mice with abnormal growths (arrowheads) projecting through their vaginas. (D and E) Uterine (Ut) and cervical/vaginal tumors (arrow) in double mutant mice. H&E sections of 5 week and 9 week old control (F, H, L)) and mutant (G, I, M) uteri. Colocalization of CK8 (green) and αSMA (red) in control (J, N and O) and double mutant (K, P and Q) uteri. Ut: uterus. Bars: 50 um.

PJS patients can develop leiomyosarcomas [46] reinforcing the importance of LKB1 in smooth muscle function and differentiation. Also, deregulation of mTORC1 signaling and mutations in various components of this pathway are commonly observed in human smooth muscle tumors [47], [48]. Paradoxically, loss of only PTEN, an upstream regulator of the mTORC1 pathway, in mesenchymal cells of the female reproductive tract is unable to induce carcinogenesis in either stromal cells or the adjacent epithelium [45], [49], [50]. Similarly, PTEN loss alone is unable to initiate polycystic kidney disease or kidney cancer in mice [51]. However, *Pten* deletion does enhance polycystic kidney disease and progression of the kidney cancer phenotype and decreases the lifespan of *Tsc1* mutant mice by over activating the mTORC1 pathway [51]. In this study we examined whether PTEN loss in mesenchymal cells synergizes with LKB1 loss by developing another mouse model with conditional deletion of both *Lkb1* and *Pten* genes (*Lkb1^cko^;Pten^cko^*). Grossly, the female reproductive tracts of 5 week old *Lkb1^cko^;Pten^cko^* mice were enlarged and showed abnormal growths compared with *Lkb1^fl/fl^;Pten^fl/fl^* controls (Figure 7A and 7B). By 9 weeks, tumorous growths were observed projecting through the vaginal opening of *Lkb1^cko^;Pten^cko^* mice (N = 8/10) but not in *Lkb1^cko/+^;Pten^cko^* or *Lkb1^fl/fl^;Pten^fl/fl^* mice (Figure 7C). Dissection of *Lkb1^cko^;Pten^cko^* double mutant mice revealed large tumorous growths in the uteri and in the cervix/vagina region (N = 20/20) (Figure 7D and 7E). Histological examination of *Lkb1^cko^;Pten^cko^* uteri showed development of endometrial intraepithelial neoplasia by 5 weeks (N = 4/4), which progressed to endometrial adenocarcinoma in 9 week old mice (N = 10/10) (Figure 7F–7M). In comparison, endometrial cancer development usually required approximately 24 weeks to be observed in *Lkb1^cko^* mice. Colocalization of αSMA and CK8 showed that, similar to *Lkb1^cko^* mice (Figure 5), expansion of αSMA+ cells adjacent to CK8+ epithelial cells was also observed in *Lkb1^cko^;Pten^cko^* mutant mice (Figure 7N–7Q). Examination of oviducts from *Lkb1^cko^;Pten^cko^* mutants showed development of oviductal cysts and adenomas akin to *Lkb1^cko^* mice (Figure S6A and S6B). However, oviductal abnormalities appeared much earlier in *Lkb1^cko^;Pten^cko^* mutants (12 weeks) compared with *Lkb1^cko^* mice (18 weeks).

Human cervical cancer and PJS patients show alterations in LKB1/mTORC1 signaling [10], [18], [52]. Even though Misr2-Cre causes recombination in stromal cells of the cervix and upper vagina (Figure S1), no tumor formation was observed in these organs of *Lkb1^cko^*, *Tsc1^cko^*, and *Tsc2^cko^* mice (N = 5/each; data not shown). However, loss of both LKB1 and PTEN initiated tumor formation in cervices and vaginas of mutant animals (N = 20/20) (Figure S6C and S6D). Histological examination of the lower reproductive tracts (endocervix, cervix and vagina) of 5 week old mice showed expansion of the stromal compartment and hyperplasia of the adjoining epithelial cells (Figure S6E and S6F). By 10 weeks, hyperplasia and/or neoplasia of cervical and vaginal squamous epithelial cells were observed in *Lkb1^cko^;Pten^cko^* mice (N = 20/20) (Figure S6H–S6J). No similar changes were observed in *Lkb1^fl/fl^;Pten^fl/fl^* control mice (N = 5) (Figure S6G). Interestingly, massive expansion of mesenchymal cells or areas with mesenchymal only features were also observed in all cervical and vaginal tumors examined (Figure S6K).

## Discussion

Mutations in *LKB1* are frequently observed in PJS patients and associated with development of gastrointestinal, colorectal, pancreatic, breast, and gynecological and gonadal cancers [5], [53]. LKB1 inhibits mTORC1 activity through AMPK, and loss of LKB1 is associated with increased mTORC1 activity in both human and murine tumors [5], [54], [55]. The mechanisms controlled by the LKB1 in reproduction and gynecological cancers have not been thoroughly investigated. However, recent studies have highlighted the importance of the genes involved in this signaling pathway in germ cell and reproductive tract biology. Oocyte-specific loss of TSC1 or TSC2 leads to premature ovarian failure and infertility in mice [56]. The deletion of *Lkb1* in the murine uterine epithelium activates mTORC1 signaling and leads to the development of endometrial cancer [9]. Recently, we showed that conditional deletion of TSC1 in the female reproductive tract initiates oviductal epithelial cell dysplasia and causes blockage of embryo transport through the oviduct leading to infertility in mice [57]. In this report we have shown that conditional deletion of *Lkb1, Tsc1*, and *Tsc2* in the mesenchymal cells leads to the development of oviductal and uterine pathologies such as endometrial cancer, indicating that dysregulated mTORC1 signaling downstream of LKB1 signaling plays an important role in pathogenesis of the observed defects (Summarized in Table S1).

The activation of mTOR signaling by dysregulated LKB1 has been associated with smooth muscle development and associated pathologies [58]. For example, intestinal polyps in human PJS patients and an *Lkb1* heterozygous mouse model show upregulation of the mTOR activity and expansion of smooth muscle compartment [58]. The deletion of *Lkb1* using smooth muscle-specific cre mice affects smooth muscle cells and leads to hyperplasia of adjoining epithelial cells, causing development of polyps similar to those observed in human patients with PJS [11]. The upregulation of mTOR signaling is also observed in human smooth muscle tumors of the uterus known as leiomyomas and similar tumors were developed in Eker rat with defective TSC2 signaling confirming that the mTORC1 pathway plays an important role in the pathogenesis of these tumors [48]. In another example, the loss of TSC1 in cardiac smooth muscle cells results in cardiac hypertrophy and death [59]. The increase in cardiac muscle mass is associated with activation of mTORC1 signaling and rapamycin treatment of these mice rescues the phenotype and leads to prolonged survival [59]. In this study, we showed that inhibition of mTORC1 signaling by rapamycin significantly suppressed tumor burden in *Lkb1* mutant mice (Figure 6), further highlighting the contribution of dysregulated mTORC1 signaling to development of *Lkb1* mutant phenotype.

We show that mutation of stromal *Lkb1* induces proliferation of adjacent oviductal and uterine epithelium (Figure 1 and Figure 5) accompanied by increased TGFβ signaling (Figure 3 and Figure S4), which is paradoxically known to mediate both tumor suppression and promotion [60]. Increased TGFβ signaling is associated with tumor promotion in pancreatic and breast cancer models [61], [62]. In contrast, loss of TGFβ signaling leads to the development of prostate and gastric carcinomas [34]. In the intestine, loss of LKB1 in mesenchymal cells causes decreased TGFβ signaling, which is associated with polyp development, suggesting that LKB1 signaling promotes TGFβ signaling [11]. In contrast, a recent study showed that LKB1 inhibits TGFβ signaling and promotes epithelial differentiation [32]. We suspect that the role of TGFβ in carcinogenesis is highly context and/or tissue dependent [34].

*LKB1* mutations have been observed in approximately 20% of human cervical cancer patients [10] and activation of mTORC1, a downstream target of LKB1 signaling, has been observed in 54% of human cervical adenocarcinoma patients [63]. Inhibition of mTORC1 decreases proliferation and induces apoptosis in cervical cancer cell lines [52]. These studies indicate the critical role played by this pathway in cervical tumorigenesis. Human PJS patients also develop malignant tumors in the endocervix known as adenoma malignum [18]. However, tumor formation in the cervix has not been reported in *Lkb1* mutant mouse models [9], [19]. Because these previous studies mainly focused on the role of LKB1 signaling in epithelial cells, the contribution of dysregulated stromal LKB1 signaling in cervical carcinogenesis has been unappreciated. Examination of the lower female reproductive tracts (cervix and vagina) collected from *Lkb1*, *Tsc1*, and *Tsc2* mutant mice showed no abnormal growth in these organs. However, cervical/vaginal epithelial hyperplasia and neoplasia were observed in *Lkb1^cko^;Pten^cko^* mice (Figure 7), indicating that loss or alterations in the activity of another tumor suppressor in combination with defective LKB1 activity is required for cervical carcinogenesis.

We have shown that defective LKB1/TSC1/TSC2/mTORC1 signaling in mesenchymal cells is sufficient to cause epithelial hyperplasia, adenoma and paratubal cysts in oviducts, and uterine endometrial cancer in mice. A significant proportion of PJS patients harbor mutations that encompass whole or partial *LKB1* gene deletions [64], and comparative analyses of human oviductal/ovarian adenomas revealed similar changes in the stromal population, making this mouse model appropriate for studying this disease. Future studies will investigate human oviductal or ovarian adenomas and endometrial carcinoma associated mesenchymal cells for genetic mutations or alterations in this signaling pathway with the expectation of strong support for treating PJS patients with therapies targeting mTORC1, such as rapamycin and its analogs.

## Materials and Methods

### Mouse genetics and husbandry

All protocols involving animal experimentation are in compliance with the NIH Guide for the Care and Use of Laboratory Animals and were approved by the Institutional Animal Care and Use Committee at Massachusetts General Hospital. Mice used in this study were housed under standard animal housing conditions and maintained on a mixed genetic background (C57BL/6;129/SvEv). The following mice strains: *Amhr2^tm3(cre)Bhr^* (also known as *Misr2-Cre*, obtained from Dr. Richard Behringer, [16]), and *Stk11^tm1Rdp^* (*Lkb1^fl/fl^*, [19]), *Tsc1^fl/fl^* [65], *Tsc2^fl/fl^* [66], *Pten^tm1Hwu^* [67] were mated to produce *Amhr2^tm3(cre)Bhr^*/*+;Stk11^Δ/Δ^*, *Amhr2^tm3(cre)Bhr^*/*+;Tsc2^Δ/Δ^*, *Amhr2^tm3(cre)Bhr^*/*+;Tsc1^Δ/Δ^*, *Amhr2^tm3(cre)Bhr^*/*+;Pten^Δ/Δ^* and *Amhr2^tm3(cre)Bhr^*/*+;Stk11^Δ/Δ^; Pten^Δ/Δ^ hereafter* referred to as *Lkb1^cko^*, *Tsc2^cko^*, *Tsc1^cko^*, and *Lkb1^cko^Pten^cko^* respectively. Whenever possible, littermate control mice were used in all experiments. *Misr2-Cre;Rosa26Lacz^fl/fl^* reporter mice were generated as previously described [4]. Tail biopsies were used to perform genotyping using standard PCR protocols as described for *Misr2-Cre* [22] and with the following primers and conditions to detect wt and flox alleles of *Lkb1* 5′-GGG CTT CCA CCT GGT GCC AGC CTG T, 5′-GAT GGA GAA CCT CTT GGC CGG CTC A-3′ and 5′-GAG ATG GGT ACC AGG AGT TGG GGC T and 35 cycles of 94 C 30 sec, 65 C 1 min, 72 C 1 min. PCR conditions for *Tsc1, Tsc2*, and *Pten* alleles are previously described in detail [45], [57], [66]. Gross pictures of were taken using a Nikon SMZ1500 microscope with an attached Spot camera (Diagnostic Instruments, Sterling Heights, MI) or with a Nikon D60 digital camera and macro lens. Bromophenol blue dye injections were performed as previously described [20]. Briefly, 0.25% bromophenol blue solution was injected into the lumen of oviducts at the uterotubal junction or lumen of the uterus in adult control and *Lkb1^cko^* mutant oviducts (N = 3/each). The oviducts were grossly examined under microscope.

*Lkb1^cko^* mutant mice (>7 month old, N = 3/group) were given either rapamycin (250 ug by oral gavage, Rapamune, Wyeth, PA) or an equivalent volume of vehicle control (The American Lecithin company, Oxford, CT) as previously described in [45] for five days per week for three weeks. After the treatment period, animals were euthanized and tissues were collected for further analyses.

### Histology, immunohistochemistry (IHC), immunofluorescence (IF), β-galactosidase staining

The female reproductive tracts from control and mutant animals were collected at different stages of development. For histological examination, tissues were fixed in 4% paraformaldehyde for 10–12 h at 4°C, processed as previously described [4], and examined by an experienced human oviductal pathologist. Paraffin embedded tissue sections of human oviductal/ovarian adenomas were obtained from Department of Pathology, Brigham and Women's Hospital using Institutional Review Board-approved protocols. Detailed methods for IHC and IF are described elsewhere [4], [68]. Tumors were graded using the FIGO staging system. The primary and secondary antibodies used in this study are: β-catenin (BD Transduction Laboratories, San Jose, CA); E-cadherin (Santa Cruz Biotechnology, Inc., Santa Cruz, CA); αSMA (Sigma, St. Louis, MO); TJP-1/ZO-1 (Developmental Studies Hybridoma Bank, Iowa City, IA); phospho-riboprotein S6, phospho-SMAD1 (Ser463/465)/5(Ser463/465)/8 (Ser426/428), phospho-mTOR (Cell Signaling Technology, Danvers, MA); phospho-SMAD2, phospho-Histone H3 (Millipore, Billerica, MA); cytokeratin, desmin (Neomarkers, Fremont, CA); cytokeratin 8 (Developmental studies hybridoma bank, IA), PAX2, AlexaFluor second antibodies (Invitrogen, Carlsbad, CA); and biotinylated donkey antimouse or antirabbit F(ab)_2_ fragments (Jackson ImmunoResearch, West Grove, PA). Photos were taken with a Nikon TE2000S with an attached Spot camera (Diagnostic Instruments) or Nikon Eclipse Ni fitted with Nikon DSF12/DS-Q1MC camera. For measurements, images were analyzed from minimum of three different animals per group using Nikon NIS elements imaging or ImageJ (National Institute of Health, Bethesda, MD) software.

For β-galactosidase staining, female reproductive tract from *Misr2-Cre;Rosa26Lacz^fl/+^* and *Rosa26Lacz^fl/+^* reporter mice were collected at 5–6 weeks of postnatal. Tissues were fixed for 1 h at 4°C then washed and stained in X-gal solution at room temperature for 3–4 h. After a quick rinse with PBS, tissues were processed for histology. Masson's Trichrome staining was performed using a kit (Sigma).

### Western blot analyses

Oviducts (N = 3/each) from *Lkb1* control and mutant animals were collected and protein extracts were prepared using RIPA buffer as described in [4]. Protein concentration was determined using the Bradford assay and equal amounts protein was loaded in gels. The following antibodies are used: S6, pS6, pRAPTOR, 4E-BP1, p4EBP1 (Cell Signaling Technology); β-catenin (Sigma); pSMAD2 (Millipore); β-actin (Neomarkers). Western blot films were scanned and bands were analyzed by pixel density for statistical analyses.

### Statistical analysis

Statistical analyses were performed using Prism software (GraphPad software, La Jolla, CA). The Student *t* test was used to calculate differences between the groups (N≥3/group), and *p* values≤0.05 were considered statistically significant.

## Supporting Information

Figure S1 Conditional deletion of LKB1 in oviductal mesenchymal cells using Misr2-Cre. (A–C) Misr2-Cre driven β-galactosidase expression (*Misr2-Cre;Rosa26LacZ^flox/flox^*) in oviductal, cervical and vaginal mesenchymal cells (S) but not in epithelial cells (Epi). Genomic PCR confirms recombination of *Lkb1* (D) and *Tsc2* (E) alleles in Misr2-Cre expressing organs (oviduct, ovary and uterus). Bars: 50 um.
(TIF)

Figure S2 Localization of cytoskeleton and epithelial proteins in mutant oviducts. 5 week old oviducts from controls (N = 3) and *Lkb1* mutants (N = 3) were examined for expression of cytokeratin (A and B), β-catenin (C and D), E-cadherin (E and F), and Tight Junction Protein 1 (TJP1/ZO-1) (G and H). Arrowheads in panel B, D, F and H point to the epithelial cells displaced into the oviductal lumen. Colocalization of PAX2 (green) and αSMA (red) in control oviducts (I and K). PAX2 expression was absent in fimbrial epithelial cells (asterisk) but present in the rest of the oviductal epithelial cells. PAX2 expression in mutant oviducts (J and L) was similar to controls. O: ovary, Ovi: oviduct, Ut: uterus Bars: 50 um.
(TIF)

Figure S3 Histological analyses of *TSC2^cko^* mutant oviducts. 6 week old oviducts from control (A and C) and *Tsc2* mutant (B and D) mice. Asterisks (*) in C and D mark the fimbriae/distal segments of the oviducts. Bars: 50 um or as otherwise mentioned.
(TIF)

Figure S4 Comparison of uteri with deletion of LKB1 in the epithelium combined with *Lkb1^cko^*. Gross female reproductive tracts from *Lkb1^fl/fl^, Lkb1^cko^*, and *Lkb1^cko/-^* mice (A–C). Arrows in panel in A to C point to the uterus. Increased expression of αSMA (D–F), pH 3 (G–I), pmTOR (J–L), and pS6 (M–O) in mesenchymal cells of 9 week old *Lkb1^cko^* uteri compared to controls. Columns represent the mean values for n as indicated. Error bars represent SEM. An asterisk indicates that expression in the mutant mice was significantly higher. White solid line outlines the area used for the analyses of αSMA- or pH 3-positive cells or brightness of pmTOR and pS6 staining. White dotted line outlines the stromal compartment or epithelial cells, which were excluded from the analyses. Co-localization of pSMAD2 and αSMA by immunofluorescence in control (P) and *Lkb1* mutant (Q) uteri. E: Uterine epithelial cells. Bars: 50 um.
(TIF)

Figure S5 Rapamycin-treated oviducts and metaplasia of the uterine epithelium in adult *Lkb1^cko^*. (A) The oviducts of *Lkb1^cko^* mice treated with rapamycin (a) have fewer and smaller cysts compared to vehicle-treated controls (b). (B) Squamous epithelial cells were observed by H&E in *Lkb1* mutant uteri (b, c, e and f) but not in controls (a and d) or *Tsc1/Tsc2* (g and h) mutants. Arrows: squamous epithelium, Arrowheads: columnar epithelium. Bars: 50 um.
(TIF)

Figure S6 Accelerated tumorigenesis in *Lkb1^cko^;Pten^cko^* mice. H&E analyses of (A) Ovary, oviduct, and uterus from 12 week old *Lkb1^fl/fl^;Pten^fl/fl^* mice and (B) oviductal cysts (arrow) and adenoma in *Lkb1^cko^;Pten^cko^* mutant mice. Lower female reproductive tract (endocervix, cervix and vagina) from 5 week old control (C and E) and mutant (D and F) mice. Cervix and vagina of adult control (G) and mutant (H and I) mice. Arrow in panel H and I mark focal stromal hyperplasia. Cervical hyperplasia and neoplasia (arrows) in *Lkb1^cko^;Pten^cko^* mice (J). Black dotted line demarcates the cervix from the uterus. Arrowhead marks focal mesenchymal cell hyperplasia. Tumors mainly consist of mesenchymal cells present in the cervix/vagina of mutant mice (K). Inset is a higher magnification image of boxed area in K. S, stroma; Ut, uterus Bars: 50 um unless otherwise indicated.
(TIF)

Table S1 Phenotype frequency.
(DOC)
