# Supplementary figures and images for: Stromal Liver Kinase B1 [STK11] Signaling Loss Induces Oviductal Adenomas and Endometrial Cancer by Activating Mammalian Target of Rapamycin Complex 1
Source: PLoS Genet. 2012 Aug 16;8(8):e1002906. doi: 10.1371/journal.pgen.1002906 (PMC3420942; doi:10.1371/journal.pgen.1002906)

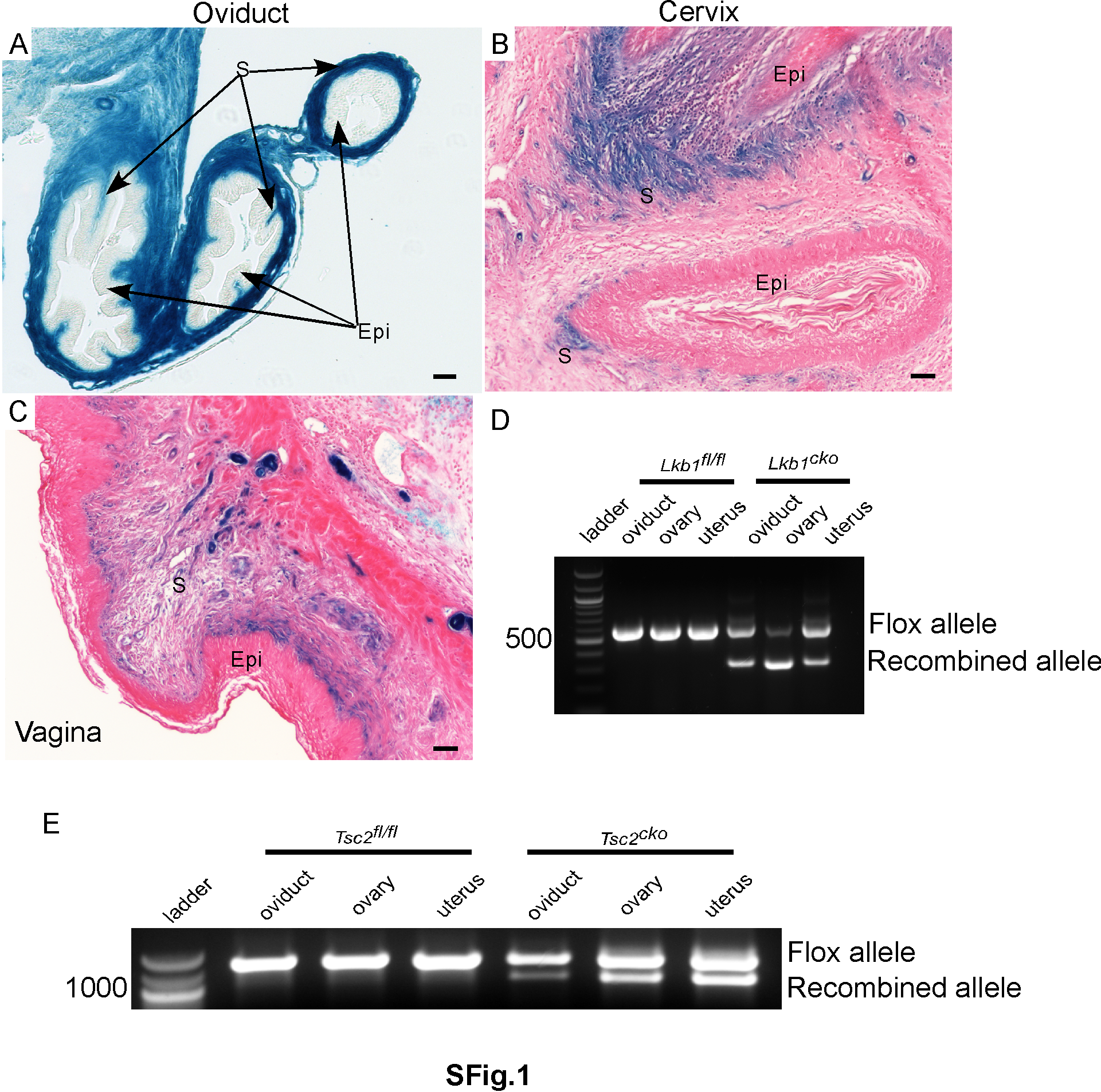

Supplement: Figure S1 — Conditional deletion of LKB1 in oviductal mesenchymal cells using Misr2-Cre. (A–C) Misr2-Cre driven β-galactosidase expression (Misr2-Cre;Rosa26LacZflox/flox) in oviductal, cervical and vaginal mesenchymal cells (S) but not in epithelial cells (Epi). Genomic PCR confirms recombination of Lkb1 (D) and Tsc2 (E) alleles in Misr2-Cre expressing organs (oviduct, ovary and uterus). Bars: 50 um. (TIF) [file pgen.1002906.s001.tif]

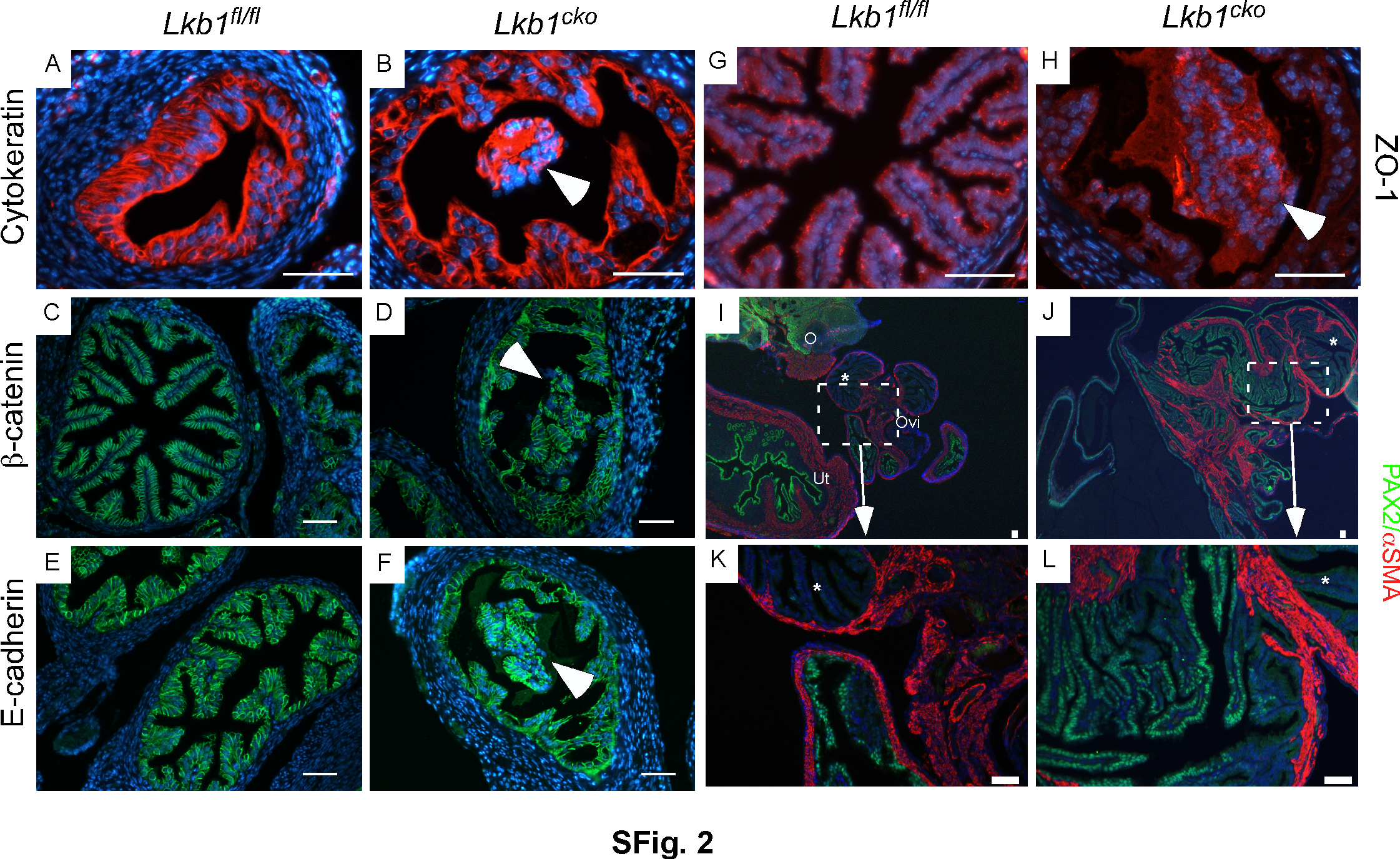

Supplement: Figure S2 — Localization of cytoskeleton and epithelial proteins in mutant oviducts. 5 week old oviducts from controls (N = 3) and Lkb1 mutants (N = 3) were examined for expression of cytokeratin (A and B), β-catenin (C and D), E-cadherin (E and F), and Tight Junction Protein 1 (TJP1/ZO-1) (G and H). Arrowheads in panel B, D, F and H point to the epithelial cells displaced into the oviductal lumen. Colocalization of PAX2 (green) and αSMA (red) in control oviducts (I and K). PAX2 expression was absent in fimbrial epithelial cells (asterisk) but present in the rest of the oviductal epithelial cells. PAX2 expression in mutant oviducts (J and L) was similar to controls. O: ovary, Ovi: oviduct, Ut: uterus Bars: 50 um. (TIF) [file pgen.1002906.s002.tif]

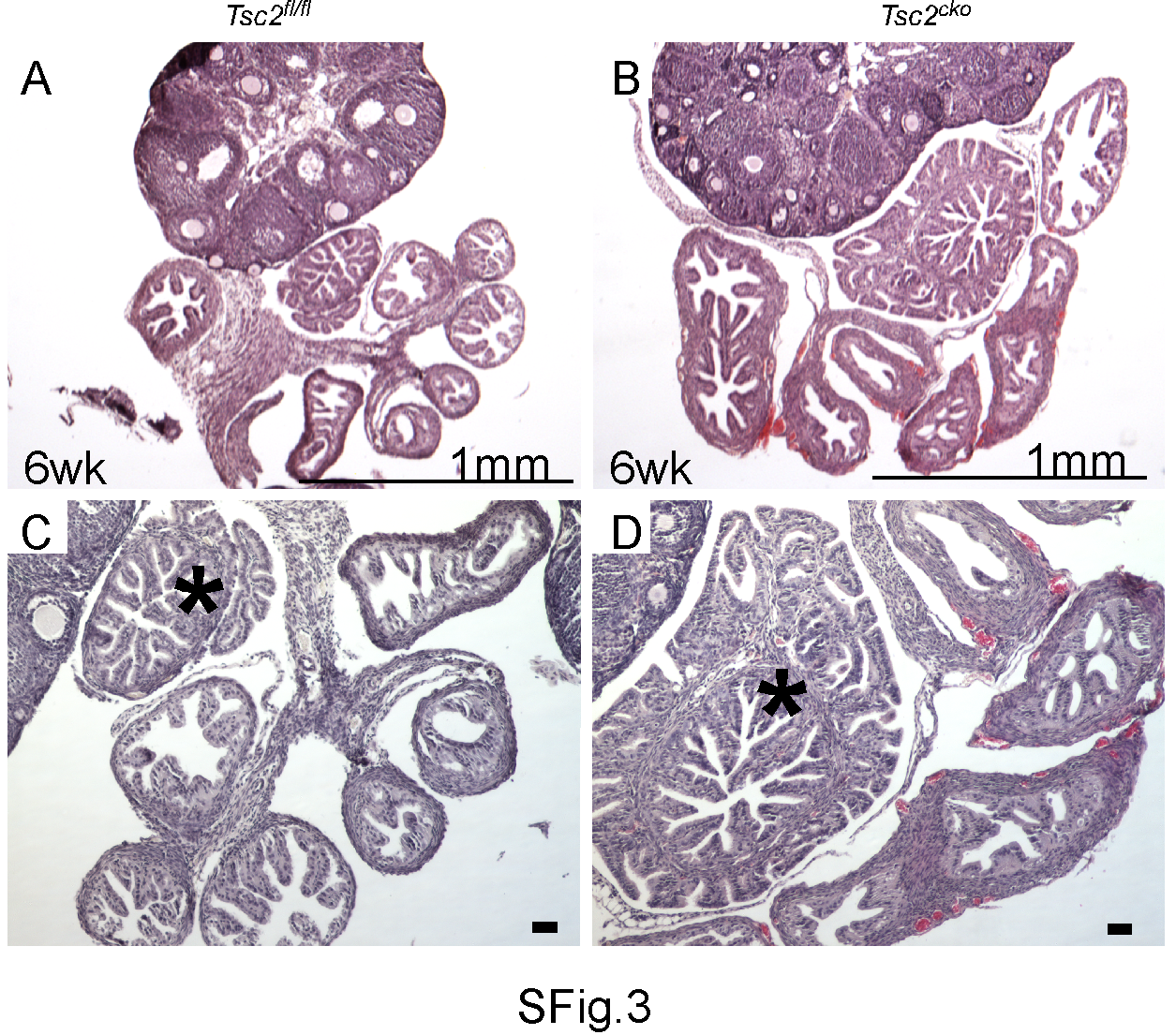

Supplement: Figure S3 — Histological analyses of TSC2cko mutant oviducts. 6 week old oviducts from control (A and C) and Tsc2 mutant (B and D) mice. Asterisks (*) in C and D mark the fimbriae/distal segments of the oviducts. Bars: 50 um or as otherwise mentioned. (TIF) [file pgen.1002906.s003.tif]

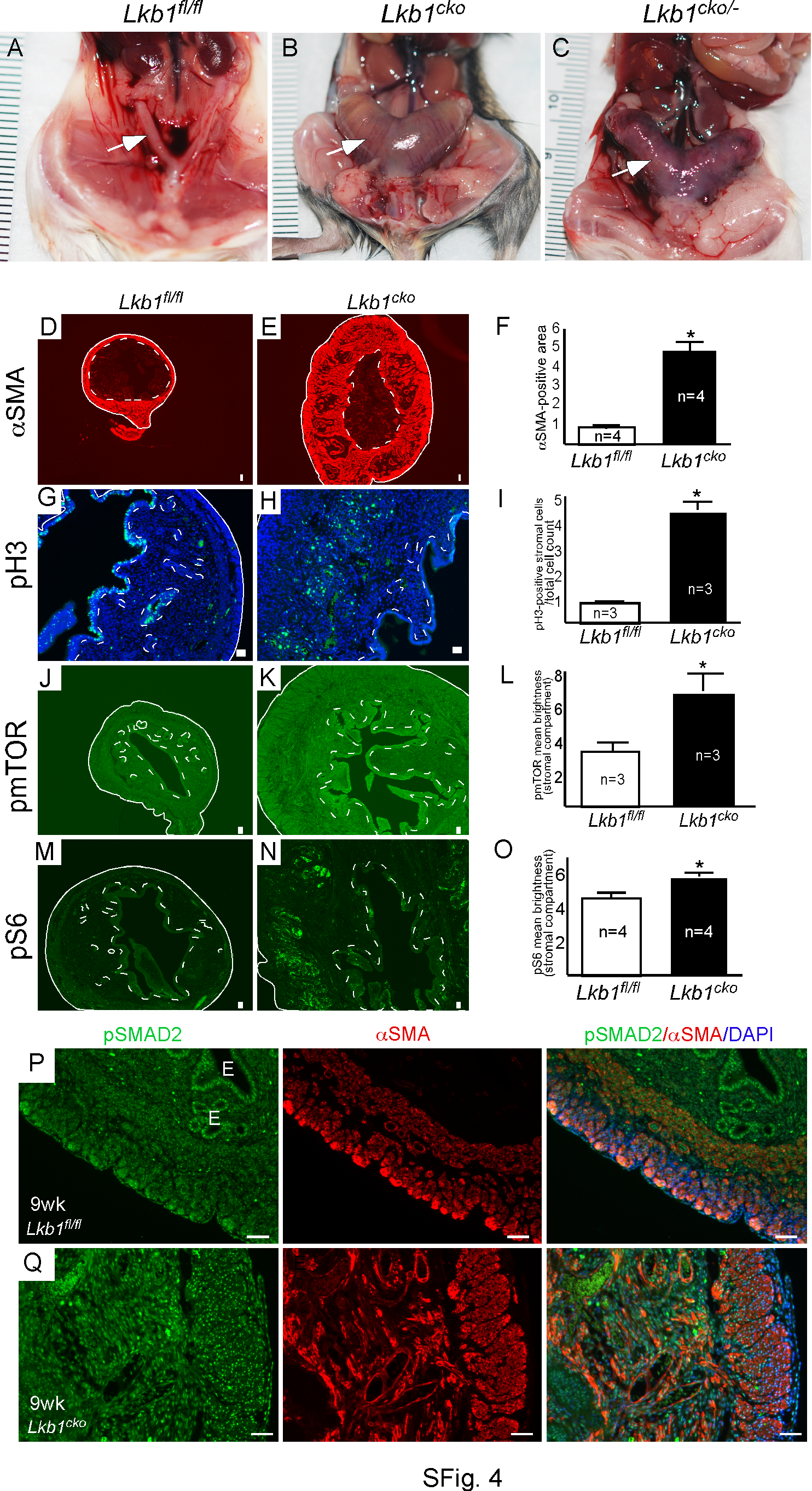

Supplement: Figure S4 — Comparison of uteri with deletion of LKB1 in the epithelium combined with Lkb1cko. Gross female reproductive tracts from Lkb1fl/fl, Lkb1cko, and Lkb1cko/- mice (A–C). Arrows in panel in A to C point to the uterus. Increased expression of αSMA (D–F), pH 3 (G–I), pmTOR (J–L), and pS6 (M–O) in mesenchymal cells of 9 week old Lkb1cko uteri compared to controls. Columns represent the mean values for n as indicated. Error bars represent SEM. An asterisk indicates that expression in the mutant mice was significantly higher. White solid line outlines the area used for the analyses of αSMA- or pH 3-positive cells or brightness of pmTOR and pS6 staining. White dotted line outlines the stromal compartment or epithelial cells, which were excluded from the analyses. Co-localization of pSMAD2 and αSMA by immunofluorescence in control (P) and Lkb1 mutant (Q) uteri. E: Uterine epithelial cells. Bars: 50 um. (TIF) [file pgen.1002906.s004.tif]

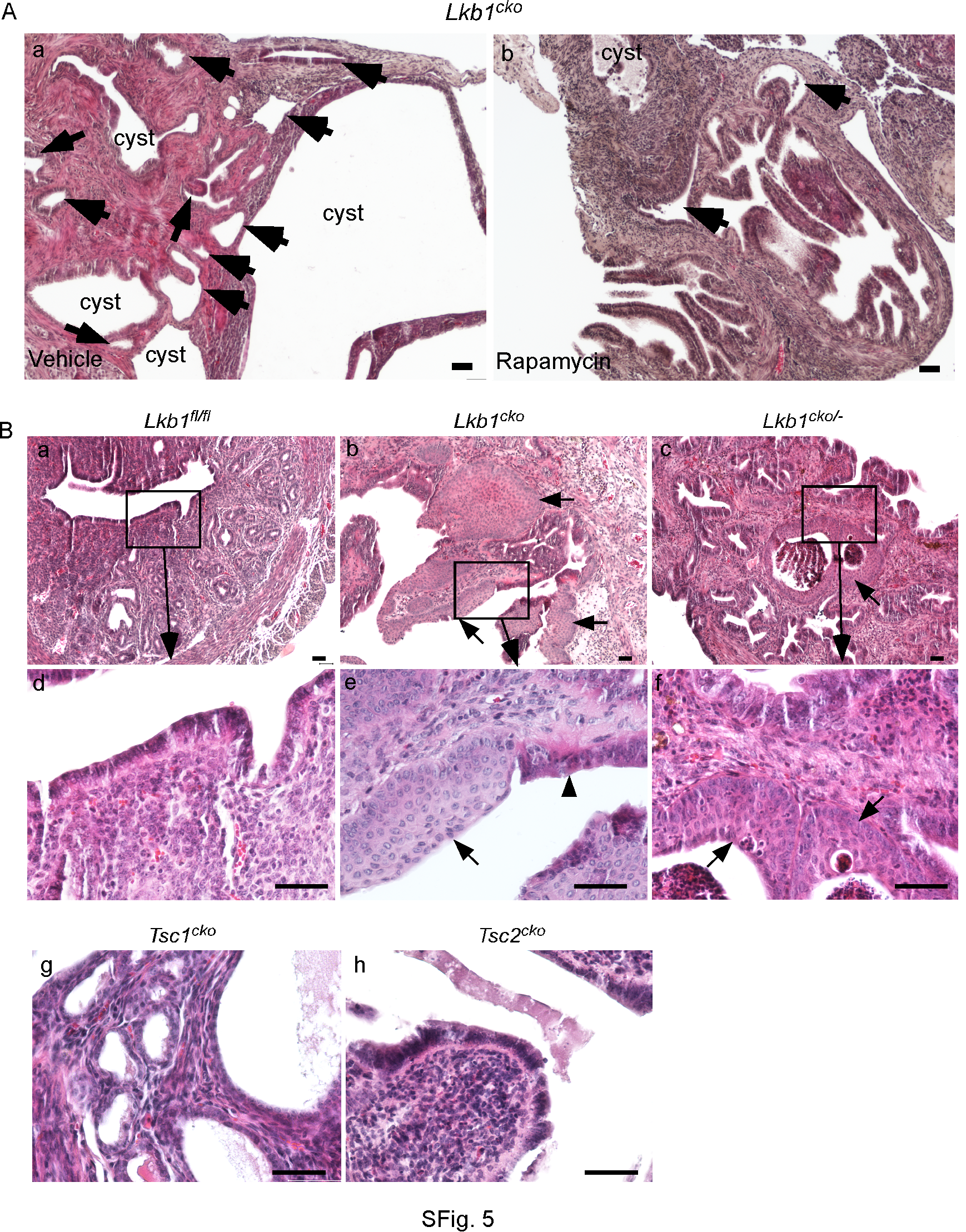

Supplement: Figure S5 — Rapamycin-treated oviducts and metaplasia of the uterine epithelium in adult Lkb1cko. (A) The oviducts of Lkb1cko mice treated with rapamycin (a) have fewer and smaller cysts compared to vehicle-treated controls (b). (B) Squamous epithelial cells were observed by H&E in Lkb1 mutant uteri (b, c, e and f) but not in controls (a and d) or Tsc1/Tsc2 (g and h) mutants. Arrows: squamous epithelium, Arrowheads: columnar epithelium. Bars: 50 um. (TIF) [file pgen.1002906.s005.tif]

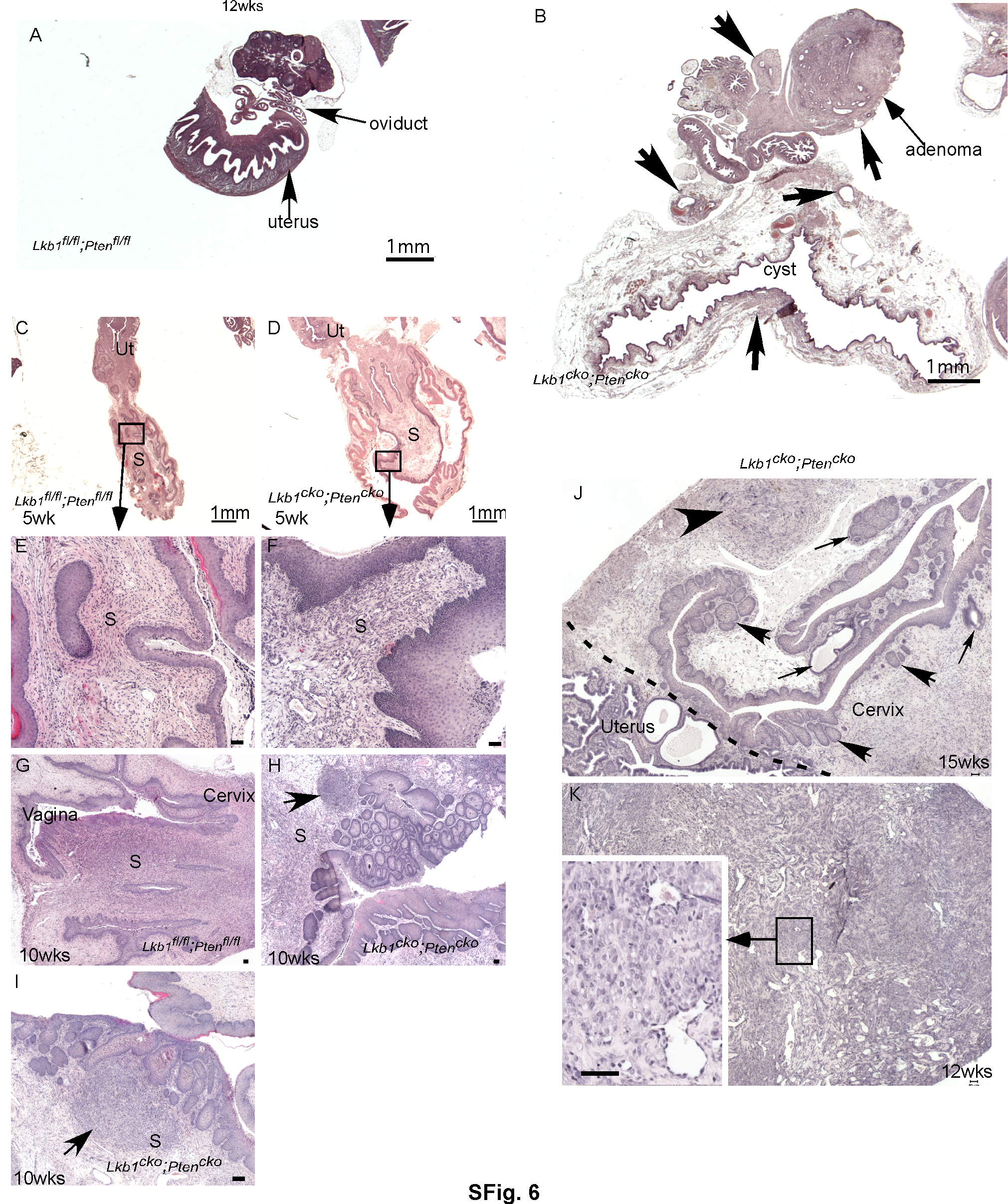

Supplement: Figure S6 — Accelerated tumorigenesis in Lkb1cko;Ptencko mice. H&E analyses of (A) Ovary, oviduct, and uterus from 12 week old Lkb1fl/fl;Ptenfl/fl mice and (B) oviductal cysts (arrow) and adenoma in Lkb1cko;Ptencko mutant mice. Lower female reproductive tract (endocervix, cervix and vagina) from 5 week old control (C and E) and mutant (D and F) mice. Cervix and vagina of adult control (G) and mutant (H and I) mice. Arrow in panel H and I mark focal stromal hyperplasia. Cervical hyperplasia and neoplasia (arrows) in Lkb1cko;Ptencko mice (J). Black dotted line demarcates the cervix from the uterus. Arrowhead marks focal mesenchymal cell hyperplasia. Tumors mainly consist of mesenchymal cells present in the cervix/vagina of mutant mice (K). Inset is a higher magnification image of boxed area in K. S, stroma; Ut, uterus Bars: 50 um unless otherwise indicated. (TIF) [file pgen.1002906.s006.tif]
