# Supplementary material for: Stromal Liver Kinase B1 [STK11] Signaling Loss Induces Oviductal Adenomas and Endometrial Cancer by Activating Mammalian Target of Rapamycin Complex 1
Source: PLoS Genet. 2012 Aug 16;8(8):e1002906. doi: 10.1371/journal.pgen.1002906 (PMC3420942; doi:10.1371/journal.pgen.1002906)
Supplement: Table S1 — Phenotype frequency. (DOC) [file pgen.1002906.s007.doc]

| **Table S1. Phenotype Frequency** | | |
| --- | --- | --- |
| **Genotype** | **Phenotype** | **Frequency** |
| ***Lkb1^fl/fl^*** | Normal female reproductive organs | 100% (N=5/5) |
| ***Lkb1^cko^*** | Oviductal cyst* and adenoma | 100% (N=5/5) |
|  | Endometrial cancer (Age:>12 weeks) | |
|  | Stage 1 | 42.8% (N=3/7) |
|  | Stage 2 | 28.5% (N=2/7) |
|  | Stage 3 | 28.5% (N=2/7) |
|  | Cervical or vaginal cancers | None (N=0/5) |
| ***Lkb1^fl/fl^;Pten^fl/fl^*** | Normal female reproductive organs | 100% (N=10/10) |
| ***Lkb1^cko^;Pten^cko^*** | Oviductal cyst* and adenoma | 100% (N=10/10) |
|  | Endometrial cancer (Age: >9 weeks) | |
|  | Stage 1 | 40% (N=4/10) |
|  | Stage 2 | 20% (N=2/10) |
|  | Stage 3 | 40% (N=4/10) |
|  | Cervical or vaginal cancers | 100% (N=20/20) |
| ***Tsc1^fl/fl^ or Tsc2^fl/fl^*** | Normal female reproductive organs | 100% (N=4/4) |
| ***Tsc1^cko^*** | Oviductal cyst* and adenoma | 100%(N=3/3) |
|  | Endometrial cancer | |
|  | Stage 1 | 100% (N=5/5) |
|  | Cervical or vaginal cancers | None (N=0/5) |
| ***Tsc2^cko^*** | Oviductal cyst* and adenoma | 100%(N=5/5) |
|  | Endometrial cancer | |
|  | Stage 1 | 100% (N=5/5) |
|  | Cervical or vaginal cancers | None (N=0/5) |

*Oviductal cysts were variable in size.
